# Cancer stem cell subsets and their relationships

**DOI:** 10.1186/1479-5876-9-50

**Published:** 2011-05-04

**Authors:** Hai-Guang Liu, Chong Chen, Han Yang, Yi-Fei Pan, Xiao-Hua Zhang

**Affiliations:** 1 Department of Oncology, The First Affiliated Hospital of Wenzhou Medical College, Wenzhou, 325000, China

## Abstract

Emerging evidence suggests that cancer stem cells account for the initiation and progression of cancer. While many types of cancer stem cells with specific markers have been isolated and identified, a variety of differences among them began to be appreciated. Cancer stem cells are hierarchical populations that consist of precancerous stem cells, primary cancer stem cells, migrating cancer stem cells and chemoradioresistant cancer stem cells, playing different roles in cancer initiation and progression. Here we propose a new concept "horizontal hierarchy of cancer stem cells" to distinguish them from vertical hierarchy cancer stem cells, cancer transient-amplifying cells and cancer differentiated cells, and summarize our current understanding of these subsets of cancer stem cells with the aim to open up novel therapeutic strategies for cancer based on this understanding.

## Introduction

Cancer is a kind of abnormal tissue that develops the ability of unlimited growth and the resistance to various survival stresses. Recently, accumulating experimental evidence supports that cancer stem cells account for the initiation and progression of cancer, which challenges the classical stochastic model of cancer development[1]. The cancer stem cell model or intrinsic model posits similar differentiation hierarchy such as hematopoietic system, cancer stem cells, cancer transient-amplifying (TA) cells and cancer differentiated cells, which is defined as vertical hierarchy here. Only cancer stem cells or cancer TA cells that reacquire self-renewal property can initiate cancer and progress into more malignant disease. However, in the stochastic model no hierarchy in cancer exists and every single cancer cell has the capacity of initiation and progression. Cancer stem cell hypothesis suggests that targeted therapy to cancer stem cells, not cancer TA cells and cancer differentiated cells, is the best measure to eradicate cancer, because traditional cancer therapies target the cancer TA cells and cancer differentiation cells, but omit cancer stem cells, thus leading to frequent cancer relapse[2].

**Figure 1:**
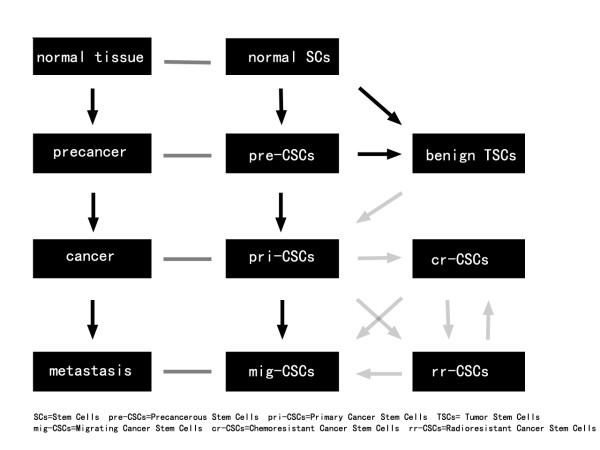
**The progression of cancer stem cells and their corresponding pathological process**. Transformed normal stem cells (SCs), progenitors with self-renewal capacity and differentiated cells after reprogramming are the potential origin of precancerous stem cells (pre-CSCs), whose corresponding pathological process is precancerous condition. Transformation from precancerous stem cells to primary cancer stem cells (pri-CSCs) is a crucial step of cancer initiation. Upon acquiring migrating capacity, primary cancer stem cells transform to migrating cancer stem cells (mig-CSCs) and metastasize to distant organs and cause metastatic cancer. In order to escape from chemoradiotherapy, some of primary cancer stem cells may develop into chemoresistant cancer stem cells (cr-CSCs) and radioresistant cancer stem cells (rr-CSCs). Some transformation steps are marked with gray arrows to indicate that they are speculative with no direct evidence up to date.

The essential features of cancer stem cells are self-renewal, multi-differentiation and tumorigenic capacity[3]. Cancer stem cells are also able to migrate and resist chemotherapy and radiotherapy. However, cancer stem cells are in constant evolution and these capacities are different among different populations of cancer stem cells. Thus we propose a horizontal hierarchy that comprises precancerous stem cells, primary cancer stem cells, migrating cancer stem cells and chemoradioresistant cancer stem cells (Figure 1). Below we will describe the horizontal hierarchy of cancer stem cells and discuss the relationship among these subsets of cancer stem cells.

## Primary cancer stem cells

Cancer cells with features of stem cells were discovered by Rudolf Virchow in the mid-19th century, who found that some cancer cells had the histological characteristics, proliferation and differentiation capacity similar to embryonic cells [4]. In 1937, Jacob Furth and Morton Kahn transplanted human leukemia cells into mice and found that the tumorigenesis of leukemia cells was different from each other. In 1960s-1970s, based on spleen-colony forming tests numerous studies showed that the tumorigenesis of cancer cells was different not only in leukemia, but also in many types of solid tumors[5-8]. Thus it is speculated that cancer, a new type of stem cell disease, was initiated from transformed stem cells and developed as a heterogeneity tissue, containing cancer stem cell subpopulations and differentiated cancer cell subpopulations.

The invention of flow cytometry greatly helped the use of specific markers to isolate subsets of cells[9]. In 1997, Bonnet et al [10] isolated two groups of leukemia cells from leukemia patients with specific surface markers CD34 and CD38, and found that CD34^+^CD38^- ^leukemia cells had the capacity of self-renewal and multi-differentiation similar to hematopoietic stem cells, and developed tumor more quickly than CD34^-^CD38^+ ^leukemia cells. Thus they concluded that CD34^+^CD38^- ^subpopulations were the initiating cells of leukemia. This was the first experimental evidence of cancer stem cells. Later, Al-Hajj et al. [11] isolated CD44^+^CD24^- ^breast cancer stem cells from breast cancer patients in 2003, thus providing the first experimental evidence of solid tumor stem cells. After that, more types of solid tumor stem cells were isolated with specific surface markers (Table 1[12-59]).

**Table 1:** Cancer stem cells with specific markers

Type of cancer	Specific markers	References
AML	CD34^+^CD38^-^Lin^-^	[10]
AML	CD123^+^	[12,13]
AML	CD47^+^	[14]
Breast cancer	CD24^-^CD44^+^Lin^-^	[11,15]
Breast cancer	ALDH1^+^	[16,17]
Brain tumors	CD133^+^	[18-20]
Glioblastoma	SSEA-1^+^	[21]
Glioblastoma	A2B5^+^	[22]
Prostate cancer	a2β1^hi^CD133^+^	[23]
Prostate cancer	Lin^-^Sca-1^+^CD49f^high^	[24]
Bladder cancer	ALDH1^+^	[25]
Lung cancer	SP-C^+^CCA^+^	[26]
Lung cancer	CD133^+^	[27]
Lung cancer	ALDH1^+^	[28]
Melanoma	CD20^+^MCAM^+^	[29]
Melanoma	CD133^+^ABCG2^+^	[30]
Melanoma	MDR1^+^	[31]
Melanoma	ABCG5^+^	[32]
Melanoma	CD271^+^	[33]
Melanoma	JARID1B^+^	[34]
Colon cancer	CD133^+^	[35-38]
Colon cancer	Lgr5^+^	[39]
Colon cancer	ALDH1^+^	[40]
Colorectal cancer	CD44^+^ESA^hi^CD166^+^	[41]
Colorectal cancer	CD26^+^	[42]
Intestinal cancer	Lgr5^+^	[39]
Intestinal cancer	CD133^+^	[43]
Pancreatic cancer	CD44^+^CD24^+^ESA^+^	[44]
Pancreatic cancer	CD133^+^	[45]
HNSCC	CD44^+^	[46]
HNSCC	ALDH1^+^	[47]
B-precursor ALL	CD34^+^CD38^+ ^CD19^+^; CD34^+^CD38^-^CD19^+^	[48]
Ovarian cancer	CD44^+^CD117^+^	[49]
Ovarian cancer	CD133^+^	[50]
Endometrial tumors	CD133^+^	[51]
Liver cancer	CD90^+^	[52]
Liver cancer	CD133^+^	[53]
Liver cancer	EpCAM^+^	[54]
Renal carcinomas	CD105^+^	[55]
Medulloblastoma	CD15^+^	[56,57]
Gastric cancer	CD44^+^	[58]
Osteosarcoma	Oct-4^+ ^	[59]

AML: acute myeloid leukaemia; ALDH: aldehyde dehydrogenase; SP-C: surfactant protein C; CCA: also known as CC10 or CCSP; MCAM: melanoma cell adhesion molecule; ABCG: ATP-binding cassette superfamily G member; MDR: multi-drug resistance protein; ESA: epithelial specific antigen; HNSCC: head and neck squamous cell carcinoma; ALL: acute lymphocytic leukaemia.

Interestingly, Xu et al [60] discovered a type of benign tumor stem cells by isolating a type of stem-like cells from pituitary adenoma with self-renewal, multi-lineage differentiation and neurospheres formation capacity. Compared with differentiated daughter cells, pituitary adenoma stem cells expressed high levels of stem cell-related proteins, anti-apoptotic proteins and pituitary progenitor markers, and had a stronger resistance to chemotherapy. Differentiation of pituitary adenoma stem cells could respond to hypothalamic hormones and secret the corresponding pituitary hormones, which were phenotypes of primary pituitary adenoma. Besides these capacities, pituitary adenoma stem cells could form tumors in the continuous xenotransplanation assays. This was the first experimental evidence of the existence of benign tumor stem cells.

At present, many types of primary cancer stem cells with specific surface markers have been isolated and the cancer stem cell hypothesis is widely accepted. However, many questions remain in the field of cancer stem cells research. For example, where primary cancer stem cells initiate from; whether primary cancer stem cells are same in the same type of cancer among different patients; and how to distinguish cancer stem cells from normal stem cells. Below, we will focus on the origin and the fate of primary cancer stem cells.

## Precancerous stem cells

Based on current literature, primary cancer stem cells may be derived from precancerous stem cells. Chen et al [61] reported the isolation of a type of precancerous stem cells from dendritic cell-like leukemic mice and the establishment of this precancerous stem cell line. The precancerous stem cells had stem cell-like phenotype, unlimited self-renewal, multi-differentiation and could reconstruct the hematopoietic system of mice after deadly radiation treatment. Transplantation of such precancerous stem cells could form tumor in immune-deficient but not in immune-competent mice. In the evolution of the tumor, the phenotype and genotype of precancerous stem cells had developed towards primary cancer stem cells.

Interestingly, Shen et al [62] discovered that the precancerous stem cells could differentiate into tumor vasculogenic progenitors and generate most of the blood vessels. Precancerous stem cells sustained the expression of vascular growth factor receptor VEGRF-2, which was under the regulation of hypoxia and various vascular growth factors such as GM-CSF, Flt3L, and IL-13, to promote vasculogenesis. In contrast, the expression of VEGRF-2 was much lower in differentiated tumor cells, indicating that vasculogenesis in precancerous stem cells is related to their inherent stem-cell characteristics.

In our opinion, precancerous stem cells have the following characteristics. First, they hide themselves in precancerous lesions. It is well known that carcinogenesis is a multi-step process. For instance, colon cancer goes through mild, moderate and severe dysplasia, adenoma, carcinoma in situ, to invasive cancer and metastasis [63]. During this long process of carcinogenesis, precancerous stem cells undergo the transformation from normal stem cells to primary cancer stem cells. Precancerous lesions progress to cancer when precancerous stem cells transform into primary cancer stem cells [64]. Second, precancerous stem cell is a mutated stem cell that highly express stemness factors such as OCT3/4, SOX2, KLF4 and therefore develops the capacities of self-renewal, multi-differentiation and resistance to chemoradiotherapy [65]. Third, precancerous stem cells are subjected to modulation by micro-environment. They can transform into malignant tumors or benign disease, mainly depending on their communication with the micro-environment [61,66].

Based on the three characteristics described above, we can distinguish precancerous stem cells from primary cancer stem cells. First is the location. Precancerous stem cells mainly exist in precancerous lesions, but primary cancer stem cells exist in primary cancer foci. For example, ductal carcinoma in situ (DCIS) is generally considered a type of precancerous lesion of breast invasive ductal carcinoma (IDC). The precancerous stem cells in DCIS stage are confined within the duct, but develop invasive capacity upon hypoxia or other stimuli, contributing to the progression of DCIS to IDC. Therefore, precancerous stem cells develop into primary cancer stem cells, and neoplastic ductal is not precanerous lesion but cancer foci [67]. Second is the genotype and phenotype. Primary cancer stem cells are derived from precancerous stem cells and exhibit some genotypes and phenotypes of precancerous stem cells, meanwhile they have their unique profiles. Castro and colleagues found that 126 genes were upregulated and 21 genes were downregulated in DCIS compared to IDC. Therefore, precancerous stem cells of DCIS exhibit different genotypes in contrast to primary cancer stem cells of IDC [68]. In addition, Ma et al. reported that the gene expression profiling of IDC was inherited from DCIS but developed distinct gene expression signatures [69]. With regard to epigenetic alternations, DNA methylation is notable. Adenomatous polyps (APs) is generally considered as precancerous lesion of adenomatous carcionoma (AdCa). The aberrant DNA methylation can be completely reversed in APs, but not in AdCa by a nonsteroidal anti-inflammatory drug celecoxib [70], suggesting the different epigenetic profilings between precancerous stem cells in APs and primary cancer stem cells in AdCa. Third is the bi-transformation. Under different micro-environment, precancerous stem cells can transform into malignant tumors or benign disease [61]. Bi-transformation is the most important characteristic to distinguish precancerous stem cells from primary cancer stem cells. Mammary intraepithelial neoplasia outgrowths (MINOs) is a mouse model of DCIS. The culture of single cells from MINOs expressed bipotential for myoepithelial and luminal differentiation and formed unique three-dimensional 'MINOspheres'. When transplanted in vivo, MINOspheres were able to form DCIS or IDC under different micro-environment [66].

The next question is the origin of precancerous stem cells. Several studies suggested that cancer initiating cells may be responsible for the development of precancerous stem cells. Wang et al [71] reported that a subpopulation of Nkx3-1 positive luminal epithelial cells was capable of self-renewal in vivo, and such a single cell was able to reconstitute prostate tissue in grafts. When the tumor suppressor gene Pten was deleted in Nkx3-1 positive luminal epithelial cells, the populations rapidly formed high-grade intraepithelial neoplasm and carcinoma after androgen mediated regeneration of the prostate. Therefore, Nkx3-1 positive luminal epithelial cells were a type of prostate stem cells and mutation of tumor suppressor genes would lead to prostate carcinogenesis.

Additionally, Barker et al [72] and Zhu et al [43] discovered crypt stem cells as the origin of intestinal cancer. They demonstrated that Lgr5 positive or prominin1 positive subpopulations were intestinal stem cells. Deletion of Apc or activation of endogenous Wnt signaling in such intestinal stem cells led to their transformation to abnormal stem cells, resulting in intestinal neoplasm. However, when the same mutations occurred in transit-amplifying cells without unlimited self-renewal capacity, the induced adenomas grew slowly and disappeared after long observation.

The malignant transformation of normal stem cells was also discovered in mesenchymal stem cells. Røsland et al [73] showed that after long term culture for 5-106 weeks, 45.8% of bone marrow derived human mesenchymal stem cells underwent spontaneous transformation. They lost differentiation potential, had increased telomerase activity, escaped senescence, demonstrated anchorage-independent growth and were capable of tumorigenesis in vivo.

Moreover, human embryonic stem (hES) cells can transform into abnormal stem cells. Werbowetski-Ogilvie et al [74] identified two variant hES cell lines (v-hESC-1 and v-hESC-2) with different features from their parents. These variants expressed higher levels of pluripotency markers Oct4 and SSEA3, less depended on exogenous growth factors, had decreased differentiation capacity in either hematopoietic or neural conditions, and had increased frequency of teratoma initiating cells, however, their teratoma cells did not metastasize to other organs upon in vivo transplantation. Therefore, variant hES cells undergo neoplastic progression and may be the origin of malignant teratoma stem cells.

Progenitor cells may be another origin of cancer initiating cells. Jamieson et al. [75] reported that during blast-crisis of chronic myelogenous leukemia (CML), granulocyte-macrophage progenitors acquired much stronger self-renewal property due to the activation of Wnt/β-catenin pathway, and expressed BCR-ABL protein and expanded imatinib-resistant CML. Later other groups confirmed these findings [76,77]. Guibal et al [78] showed that in a murine model of acute promyelocytic leukemia (APL), a population of committed myeloid cells (CD34^+^, c-kit^+^, FcγRIII/II^+^, Gr1^int^) demonstrated enhanced self-renewal capacity through the down-regulation of the transcription factor CCAAT/enhancer binding protein-α(C/EBP-α) and were capable of efficiently generating leukemia in recipient mice. Krivtsov et al [79] reported a more detailed overview of the transformation from committed progenitor to cancer stem cells.

Self-renewal is the most essential feature of normal stem cells and cancer stem cells[80]. Notably, some mature differentiated cells can re-acquire self-renewal capacity after reprogramming and thus may be additional origin of tumor initiating cells. Takahashi and Yamanaka [81] reported that they could reprogramme mouse fibroblasts into induced pluripotent stem (iPS) cells by introducing four factors Oct3/4, Sox2, c-Myc and Klf-4. In vivo transplantation assay demonstrated that the iPS cells were able to form teratomas and it was speculated that the two oncogenes c-Myc and Klf-4 might endow iPS cells with the capacity of tumorigenesis. More recent studies demonstrated that iPS cells could be induced from differentiated cells by chemicals or proteins without the use of viral vectors[82-89]. These iPS cells with capacity of tumorigenesis might be another origin of malignant teratoma stem cells.

Taken together, adult stem cells, embryonic stem cells, progenitors with unlimited self-renewal capacity, and induced pluripotent stem cells are the potential origins of cancer initiating cells.

## Migrating cancer stem cells

Metastasis is a very important feature of malignant tumors, accounting for 90% death of tumor patients[90]. Metastasis is a multi-step process that involves progressive growth, vascularization, invasion, detachment, embolization, survival in the circulation, arrest, extravasation, evasion of the host defense and progressive growth[91]. Given its complicated nature, metastasis is far from being understood completely and many hypotheses have been proposed to elucidate the underlying mechanisms. In seed and soil theory it is speculated that metastasis is closely related to the characteristics of tumor types and metastatic sites. Different tumor cells tend to move to their specific distant organs, and different distant organs tend to accept specific tumor cells[91]. In 1980, Hart and Fldier [92] transplanted lung, ovarian and kidney tissues into subcutaneous and muscle of C57BL/6 mice, and then transplanted B16 melanoma cells into these mice after these transplanted tissue survived. They found tumor formation in the transplanted lung and ovarian but not kidney tissues. Importantly, there was no significant difference in the number of melanoma cells throughout the lung, ovarian and kidney tissues. This ruled out the influence of tumor cell numbers and further confirmed that metastasis is related with special distant organs.

According to cancer stem cell hypotheses, cancer stem cells are ideal seeds of metastasis. Stem cells are indeed ideal carrier of gene mutations and their accumulation. First, the initiating cell must be a cell with extensive divisions and the mutations will be not lost after several divisions. Second, the initiating cell must have long life with strong resistance to different external stress. In contrast, a mature differentiated cell is subject to senescence and death and can not be the initiator of cancer.

But not all cancer stem cells have the characteristic of migration. Hermann et al [45] reported that CD133^+^CXCR4^+ ^subsets determined the migrating phenotype of pancreatic cancer, although both CD133^+^CXCR4^+ ^and CD133^+^CXCR4^- ^pancreatic cancer stem cells could form pancreatic cancer when transplanted into athymic mice. An inhibitor of CXCR4 could significantly reduce the metastasis in group CD133^+^CXCR4^+ ^mice. Furthermore, removal of CD133^+^CXCR4^+ ^subset from CD133^+ ^cancer stem cells could disrupt the metastasis of pancreatic cancer, but did not affect tumorigenesis in primary organ. Collectively, these data suggest that CD133^+^CXCR4^+ ^cancer stem cells determine the metastasis and represent the migrating cancer stem cells of pancreatic cancer.

Furthermore, Yang et al [52] reported that CD90^+ ^but not CD90^- ^liver cancer cells were able to form tumor. Notably, CD90^+^CD44^+ ^subpopulations had stronger capacity of tumorigenesis and metastasis than CD90^+^CD44^- ^subpopulations, and the proportion of CD90^+^CD44^+ ^subpopulations in metastasis increased compared to primary cancer. Therefore, CD90^+^CD44^+ ^subpopulations might be the migrating cancer stem cells of liver cancer.

However, current studies on migrating cancer stem cells are very limited, mainly due to the lack of specific migrating markers to isolate migrating cancer stem cells from primary cancer stem cells. It has been established that epithelial to mesenchymal transition (EMT) is involved in migration and metastasis, thus providing some clues on how to isolate migrating subpopulations from primary cancer stem cells. Mani et al [93] isolated CD44^low^CD24^high ^and CD44^high^CD24^low ^subpopulations from five breast cancer tissues and applied serial analysis of gene expression to reveal that CD44^high^CD24^low ^subpopulations expressed high level of mesenchymal markers N-cadherin, Vimentin, Fibronectin, Zeb2, Foxc2, Snail, Slug, Twist1 and Twist2, and low level of E-cadherin. They further transplanted human mammary epithelial cells constitutively expressing either Snail or Twist into immune-deficient mice and found that both of them had more efficiency of tumorigenesis, and the number of CD44^high^CD24^low ^subpopulations is elevated. Therefore, they concluded that EMT might be responsible for the generation of migrating cancer stem cells.

Zhang et al. [94] discovered that in three-dimensional culture, epithelial growth factor receptor tyrosine kinase inhibitor erlotinib inhibited the motility of inflammatory breast cancer (IBC) cell line SUM149 and its invasion in matrigel, accompanied with increased expression of E-cadherin and reduced expression of vimentin and β-catenin. Furthermore, they transplanted SUM149 cells into athymic nude mice and demonstrated that erlotinib inhibited the growth of tumor and lung metastasis by regulating the expression of E-cadherin and vimentin. This study suggests that erlotinib reversed EMT of IBC to inhibit metastasis. In this aspect, it is important to note a few of molecule implicated in both EMT and stemness such as Six1[95,96] and p21CIP1[97].

Based on these studies it is a potential approach to utilize mesenchymal markers to isolate migrating cancer stem cells from primary cancer stem cells.

## Other subsets of cancer stem cells

One significant feature of cancer is its relapse after chemotherapy and radiation. This is because a few of cancer cells evolve with the capacity of resistance to chemotherapy and radiation. Whether primary cancer stem cells can evolve into chemoradioresistant cancer stem cells is not well known but recent studies provided indirect evidence for the existence of chemoradioresistant cancer stem cells.

## Chemoresistant cancer stem cells

Todaro et al [98-100] reported a subpopulation of human colon cancer stem cells resistant to the most popular chemotherapeutic agent oxaliplatin or 5-fluorouracil (5-FU) at clinically relevant doses. Mechanistically, in this subpopulation interleukin-4 (IL-4) is produced in an autocrine manner to induce the expression of the antiapoptotic proteins cFLIP, Bcl-xL, and PED. The antagonist of IL-4 combined with oxaliplatin or 5-FU could effectively inhibit the growth of these cancer stem cells in vitro and in vivo, and decrease the size of spheroid and tumor.

ATP-binding cassette superfamily is one type of multi drug resistant proteins, which can pump chemotherapy drugs out of the cell and lead to chemoresistance[101,102]. ABCG2 is a member of this family and represents a purified marker of cancer stem cells [103]. However, targeted therapy with ABCG2 antagonist can only inhibit partially the growth of SP cells and cancer stem cells. This may be because cancer stem cells express other drug resistant proteins such as ABCB1[104].

Despite these reports demonstrating the relationship between cancer stem cells and chemoresistance, further studies are crucial to provide direct evidence supporting the existence of chemoresistant cancer stem cells, which may help develop alternative strategy for chemotherapy and targeted therapy.

## Radioresistant cancer stem cells

Diehn et al [105] reported that human and mouse breast cancer stem cells had lower levels of reactive oxygen species (ROS) than their non-tumorigenic progeny. Moreover, human cancer stem cells contained higher levels of antioxidant defense systems and developed less DNA damage after ionizing radiation, compared with non-tumor cells. Therefore, the heterogeneity of ROS levels in cancer stem cell subsets might contribute to their radioresistance. In addition, in CD133 positive glioma stem cells the expression of the autophagy-related proteins LC3, ATG5 and ATG12 was increased as a response to γ-radiation[106]. Glioma stem cells and breast cancer stem cells could also escape from radiotherapy through preferential activation of the DNA damage response [106,107]. However, whether primary cancer stem cells contain a population of radioresistant subset remains unclear.

## Relationships among cancer stem cell subsets

Up to now, precancerous stem cells, primary cancer stem cells and migrating cancer stem cells have been proven to exist in the progression of cancer [45,52,61,62], while direct experimental evidence for the existence of chemoradioresistant cancer stem cells is still required. Based on current literature, precancerous stem cells may be originated from normal stem cells, progenitors which acquire unlimited self-renewal, or differentiated mature cells after reprogramming. They may exist in precancerous lesions and are able to transform into primary cancer stem cells or benign tumor stem cells depending on the microenvironment. While benign tumor stem cells may be originated from normal stem cells and become the driving force of growth and progression of benign tumor, it remains unknown whether benign tumor stem cells can be transformed into primary cancer stem cells (Figure 1).

Primary cancer stem cells may play the most important role in the progression of cancer and recurrence. Thus the transformation from precancerous stem cells to primary cancer stem cells is a crucial step in tumorigenesis. When primary cancer stem cells acquire migrating capacity through different mechanisms such as EMT, they metastasize to distant organs and cause metastatic cancer. Therefore, migrating cancer stem cells may be originated from primary cancer stem cells and this transition may be a key step of metastasis. In order to escape from chemoradiotherapy, primary cancer stem cells may develop into chemoradioresistant subsets, which is an important reason of chemoradioresistance and cancer recurrence after traditional chemotherapy and radiation therapy. Whether chemoradioresistant cancer stem cells can transform into migrating cancer stem cells is still not known.

## Conclusion

In summary, based on the above discussion we propose the model shown in Figure 1 to demonstrate the relationship among the different subsets of cancer stem cells and their relevance to the pathological process of tumorigenesis. Undoubtedly, our deeper understanding of cancer stem cells subsets may help validate this model and open up novel therapeutic strategies for cancer. For example, we may attack migrating cancer stem cells to eliminate cancer metastasis, or eradicate chemoradioresistant cancer stem cells to overcome the resistance to chemotherapy and radiation therapy.

## Conflicts of interests

The authors declare that they have no competing interests.

## Authors' contributions

LHG, CC, YH, PYF, ZXH all contributed to the development of the concept, literature review, discussions, and writing of the manuscript. All authors have read the manuscript and agree to its submission.
